# Supplementary material for: MetaRibo-Seq measures translation in microbiomes
Source: Nat Commun. 2020 Jun 29;11:3268. doi: 10.1038/s41467-020-17081-z (PMC7324362; doi:10.1038/s41467-020-17081-z)
Supplement: Supplementary file 10 — Supplementary Data 7 [file 41467_2020_17081_MOESM10_ESM.zip › File2/Confidence_High_Taxonomy/368127_out.krona.html]

Javascript must be enabled to view this page.

members
magnitude
magnitudeUnassigned
count
unassigned
taxon
rank

368127\_out

3

3
superkingdom
2

976
3
phylum

200643
class
3

171549
order
3

3
family
171552

3
genus
838


SRS012969\_contig\_number\_10910SRS023526\_contig\_number\_37069SRS077849\_contig\_number\_21693
2292365
3
species
